# When Silence Isn’t Golden: The Case of “Silent” Atrial Fibrillation

**DOI:** 10.19102/icrm.2017.081102

**Published:** 2017-11-15

**Authors:** James A. Reiffel

**Affiliations:** ^1^Department of Medicine, Division of Cardiology, Electrophysiology Section, Columbia University, New York, NY, USA

**Keywords:** Atrial fibrillation, implantable cardiac monitor, silent atrial fibrillation, subclinical atrial fibrillation, undetected atrial fibrillation

## Abstract

Silent atrial fibrillation (AF) is common. In some patients, it is the only manifestation of AF, while in others, the AF may be symptomatic or both symptomatic and asymptomatic. Regardless, however, to date, the significance, detection, and management considerations for silent AF have been incompletely elucidated. This current study aimed to review, for both the current clinician and investigator, considerations and attitudes and the ongoing studies, respectively, with respect to silent AF. The methods used were a literature review and personal trial and clinical experience; the frequency of silent AF, concerns regarding silent AF, methods to detect silent AF, and prospective trials focused on the detection and management of silent AF were considered. The results of the literature search indicated that recently conducted relevant trials, such as PREDATE AF, ASSERT-II, and REVEAL AF, have shown that silent AF is frequent in patients with risk markers for AF and stroke in whom no prior AF history is present, and in whom no pacemaker or implantable cardioverter-defibrillator implantations have been previously performed. Furthermore, the GLORIA-AF Registry has reported the observance of more permanent AF and more prior strokes in asymptomatic patients. Ongoing trials such as ARTESiA and NOAH-AFNET 6 are expected to clarify the benefits and risks of oral anticoagulation in patients with silent AF. At present, when silent AF is detected in patients with stroke risk markers, most practitioners initiate an anticoagulation regimen.

## Introduction

“If speech is silvern, then silence is golden.” According to *Wise Words and Wives’ Tales: The Origins, Meanings, and Time-Honored Wisdom of Proverbs and Folk Sayings Olde and New* by Stuart Flexner and Doris Flexner (Avon Books, New York 1993), this phrase was first stated in a proverb in the Judaic biblical commentaries called the “Midrash” (ca 600).

While we can debate whether it is more often preferable to be verbally vocal or silent, can we say the same about atrial fibrillation (AF)? Although some patients are aware of each and every episode of AF, many are aware of only some, and some are even aware of none. The latter is commonly referred to as “silent AF” (also sometimes called subclinical or undetected or unrecognized AF). Importantly, though AF guidelines exist for rate and rhythm control, the use of anticoagulation, and for other aspects of optimal clinical care,^1^ they are not limited only to symptomatic AF. Specifically, a requirement of symptoms is not part of the guidelines-stated treatment algorithms regarding anticoagulation. Equally important, the issue of silent AF is one with major consequences, as heart failure, stroke, systemic embolism, and death may be clinical consequences of previously undiagnosed AF.^2^ Likely, their risk relates not only to the burden of AF, but also to the type and severity of associated conditions. Accordingly, significant morbidity and occasionally mortality may result under the circumstances of undiagnosed AF and the consequent lack of prophylactic treatment interventions proven to reduce AF’s adverse consequences. Early detection of undiagnosed AF in high-risk patients may therefore allow for earlier initiation of lifesaving/life-improving therapy. This manuscript will review the issue of silent AF, its significance, its detection, and its management considerations for the interested clinician and investigator. In it, the term silent AF is used in its broadest sense—that is, cases in which AF, though present, is either unrecognized, or it is known but is asymptomatic and therefore not detected by the patient when it occurs. Finally, one caveat: among silent AF, some might include persistent AF that is not recognized by the patient until he/she realizes following cardioversion that their quality of life has improved, and that they did have some low-level symptoms that had been unappreciated.^3^ The latter are not the patients this manuscript discusses; nonetheless, the same concerns regarding prophylactic anticoagulation and rate control are equally applicable to them.

## Prevalence of silent AF

In patients with known AF, silent AF is not uncommon. At least one-third of patients with symptomatic AF are found to also have asymptomatic episodes when they are monitored,^4–6^ with some reports noting that up to 70% of episodes are silent. Silent AF episodes are commonly shorter and slower than symptomatic episodes in patients who have both, yet paroxysmal silent AF has been shown via the monitoring capabilities of implanted devices to be capable of lasting 48 hours or longer.^7^ In AFFIRM, 12% of AF patients were asymptomatic at baseline, where, compared with symptomatic patients, silent AF was seen more often in men with a longer duration of AF, a lower maximum heart rate during AF, and better LV function.^8^ In some cases, silent AF is only recognized after the patient presents with a consequence, such as heart failure or thromboembolism.^9^ Following catheter ablation for AF, post-procedure monitoring shows that the percentage of recurrent AF that is silent, rather than symptomatic, increases.^10,11^ Modeling studies, such as that by Turakhia et al.,^12^ have estimated a high burden of clinically significant silent AF. The global burden is unknown. Finally, the determined prevalence of silent AF is directly related to the intensity of monitoring: the more prolonged the monitoring, the more silent AF becomes apparent. Furthermore, symptoms alone do not provide an accurate assessment of the presence of AF or of its quantity (burden).^12,13^

## Concerns regarding silent AF

**Figure 1:**
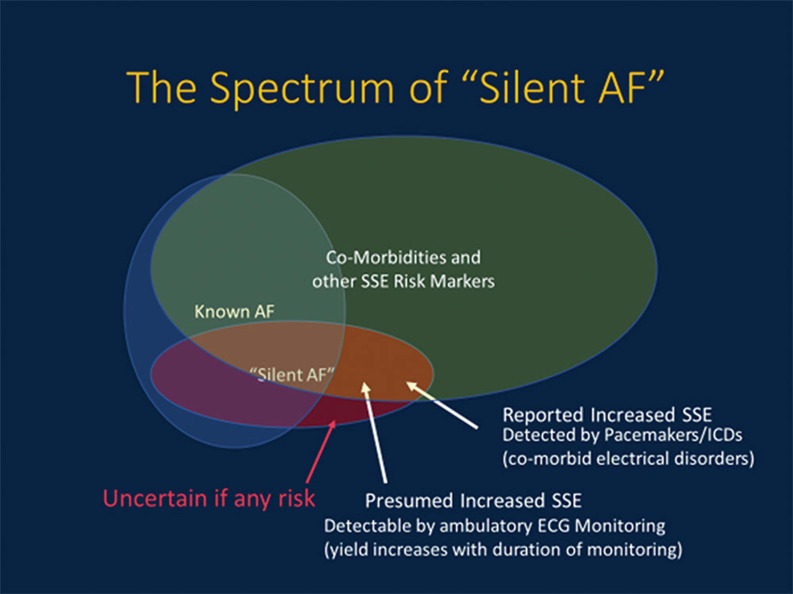
The spectrum of silent AF. Silent AF can occur in patients with known symptomatic AF or in those without other AF; in patients with comorbidities and in those without; has an apparent prevalence that increases as the duration of monitoring increases; and appears to carry a risk for stroke and systemic embolism in parallel with symptomatic AF.

The concerns regarding silent AF are similar to those associated with symptomatic AF, aside from the addition of impaired quality of life that symptoms from AF may produce. That is, both symptomatic and silent AF may result in heart failure^14^ or stroke in appropriate clinical circumstances **(Figure 1),** while with regards to silent AF specifically, the onset may be more acute or unanticipated or the incidence higher because unrecognized silent AF is less apt to have resulted in a rate control or oral anticoagulant having been prophylactically prescribed. Moreover, and very importantly, atrial thrombogenesis does not automatically accompany patient awareness of AF. The atrial alterations that can be prothrombotic, including atrial dilation, altered histology, impaired contractile and endothelial function, and stasis, are dependent upon the presence of AF and changes that may be resultant from age and/or comorbidities, but are not dependent upon the patient having palpitations, fatigue, or other AF-related symptoms. Accordingly, symptoms are not part of the risk-scoring systems for stroke, such as CHADS_2_ or CHA_2_DS_2_-VASc, and the 2014 American Heart Association/American College of Cardiology/Heart Rhythm Society Guidelines for the Management of Patients with Atrial Fibrillation specifically state that when balancing the risks and benefits when selecting an antithrombotic regimen, that “AF, whether paroxysmal, persistent, or permanent, and whether symptomatic or silent” should be considered.^1^ The GLORIA-AF Registry has also reported the occurrence of more permanent AF and twice as many prior strokes in patients with asymptomatic AF than in those with AF-related symptoms.^15^

The risk of thromboembolism with AF appears to be related both to the type and severity of the associated disorders, and to the duration of the periods of AF (the AF burden).^16,17^ Notably, Botto et al.^16^ demonstrated that the higher the CHADS_2_ score was, then less AF was required to result in thromboembolic events. The interplay between the AF, any associated disorders, and their combined effect on the left atrium (LA) also provides an explanation for the observation that stroke in patients with AF is not necessarily directly synchronous with the time at which AF is present or terminates. The abnormal atrial milieu that underlies thrombogenesis and embolism may still be present when an episode of AF ends if the abnormalities produced by the comorbidities and by the overlying atrial tachycardiac myopathic changes induced by the AF itself are still present and significant.

The fact that silent AF is associated with an increased risk for thromboembolism has perhaps best been demonstrated to date by studies that have been performed involving patients with implanted pacemakers or implantable cardioverter-defibrillators (ICDs), and in studies of patients with cryptogenic stroke.^18–27^ It is important to recognize, though, that patients with pacemakers or ICDs typically have electrical disorders other than AF, and thus that the reported incidence of silent AF and its epidemiological outcome risks may not be identical in patients without these associated disorders.

Multiple reports exist that document pacemaker/ICD-detected silent AF occurring in patients with no prior AF history; reports that associate silent AF in pacemaker/ICD patients with an increased incidence of stroke also exist.^18–24^ In the latter, the stroke risk has been associated with relatively modest amounts of AF such that AF durations of five to six minutes, 3.8 to 5.5 hours, or of 24 hours have been associated with increased hazard ratios for thromboembolism of 2.2 to 9.4.^18–24^ Of note, in a subgroup analysis of one of these, the ASSERT study, the stroke risk was significant only with an AF duration of at least 24 hours, but was not significant with either an AF duration of six minutes to six hours, or six hours to 24 hours.^28^ Importantly, however, the cited trials vary in their threshold criteria for AF, the demographics of their study populations, the duration of follow-up, and other critical characteristics that make them difficult to compare directly. Also, in ASSERT, the CHADS_2_ score was only 2.2, whereas in patients with a higher risk score a lesser AF burden may be enough to trigger clot formation.^16,17^ Nonetheless, taken together, the link between silent AF and stroke seems consistent.

Corroborating evidence relating silent AF and stroke is available from studies conducted involving stroke patients in whom AF was first reported at the time of the stroke, and in patients with cryptogenic stroke in whom long-term monitoring after the stroke demonstrated a substantial incidence of silent AF during follow-up.^24–27^ While in the latter, it is possible that the AF could just be a bystander event, rather than being causally linked, the causality is clearer when previously unknown AF is present in a patient who is admitted with acute stroke. The causality is also strongly suggested by observations that anticoagulation of patients with monitoring-detected AF post stroke has reduced recurrent stroke in patients in whom prolonged monitoring was performed, as compared with in those who underwent shorter periods of monitoring and in whom AF was detected less frequently.^29^ The Find-AF_randomized_ study showed that more prolonged monitoring detected more silent AF, which led to more anticoagulation use, which was associated with a 50% reduction in the rate of recurrent ischemic stroke.^29^

## Methods and duration of monitoring for silent AF

It is abundantly clear from the medical literature that the longer patients with intermittent arrhythmias are monitored, the greater the arrhythmia documentation will be. Thus, ambulatory monitoring is superior to random electrocardiograms (ECGs), and prolonged monitoring is superior to 24 to 48 hours of Holter monitoring.^30^ For example, in a review of monitoring reports of 600 patients randomly selected from over 100,000 tracings from a commercial monitoring database (Lifewatch, Inc., Philadelphia, PA, USA), my colleagues and I reported that clinically important arrhythmias were detected by 24-hour Holter monitoring in 6.2%, versus in 36% using 30-day auto-triggered memory loop recording.^30^ Additionally, in that data set, 24-hour Holter monitoring detected AF (including both symptomatic and silent) in 27 patients, as compared with in 146 patients with 30-day recordings.^30^ Similarly, the FIND-AF investigators presented data showing that three 10-day Holter recordings in post-stroke patients detected AF in 13.5% by six months, as compared with 4.5% in routinely assessed patients.^29^ Additionally, the EMBRACE investigators reported AF lasting at least 30 seconds in 16.1% of cryptogenic stroke patients with a 30-day event-triggered recorder versus 3.2% of patients in the control group, while the CRYSTAL AF investigators reported cumulative AF detection rates of 3.7%, 8.9%, 12.4%, and 30.0% at one, six, 12, and 36 months using an implantable loop recorder (ILR) versus a rate of 3.0% at 36 months in their control group.^26,27^ Moreover, in monitoring studies assessing AF with ILRs, the median time to first detection has routinely been longer than 30 days. Recently, smartphones have also begun to be utilized to detect AF. However, while they may be useful as alternatives to older patient-applied recorders at the time of symptomatic periods, or as a means to detect long-lasting paroxysms of AF, because of the brief and intermittent nature of their monitoring capabilities they are not optimal options for the detection of silent AF in the overwhelming majority of patients who are at risk, or for adequately evaluating AF burden, and have yields that do not compare with those of the prospective trials discussed below that utilized insertable monitors.^31^

## Prospective studies of silent AF

Notably, device-detected silent AF is not limited to studies in patients with pacemakers/ICDs or post-stroke patients. Recently, three prospectively performed trials have been reported that considered patients with risk markers for AF and for stroke who demonstrated no prior history of AF, incorporating long-term monitoring (in years) with an implanted/inserted cardiac monitor (ICM) **(Table 1)**. They are PREDATE AF, ASSERT-II, and REVEAL AF.^32–34^ Additionally, a larger but similar in nature trial, LOOP (NCT02036450), is still ongoing, while another small trial, GRAF-AF (NCT01461434), has not yet been presented or published.

### PREdicting Determinants of Atrial Fibrillation for Therapy Elucidation in Patients at Risk for Thromobembolic Events (PREDATE AF)

PREDATE AF^32^ was an investigator-initiated, prospective, single-arm, open-label, single-center trial with a planned enrollment quota of 350 asymptomatic subjects with a CHA_2_DS_2_-VASc score ≥ 2 and no history of AF who were monitored using ICMs. However, it was stopped prior to enrolling the full 350 subjects, as it met its power analysis goals early. The primary outcome was the new onset of AF ≥ six minutes in duration, as evaluated by a review of monthly ICM transmissions. Secondary outcomes included the time to first detection of AF, the comparison of AF incidence by gender, the comparison of AF incidence in subjects with high versus low CHA_2_DS_2_-VASc scores, and the characterization of treatment patterns of patients found to have AF. Ultimately, 249 patients were enrolled, with 245 (98.3%) included in the final study analysis (three patients withdrew from the trial and one was explanted to allow them to undergo radiation therapy for pulmonary cancer). During the study, three patients died due to non-cardiac causes; their data were included through their last monthly transmission. Additionally, five patients required pacemaker implantation during the course of the trial; these individuals continued in the trial per design. The mean age was 74.3 years, the mean CHA_2_DS_2_-VASc score was 4.6, the mean left ventricular ejection fraction was 81%, and 41% of the study subjects were female. The use of an antiarrhythmic drug at the time of enrollment was an exclusion criterion, but the use of β-blockers was allowed and were being taken by 61%. Anticoagulants were not taken at baseline, but 83% of subjects were using aspirin. The most common comorbidity was hypertension (over 95%), and about 20% had a remote stroke history.

During an average follow-up of 451 ± 185 days, a total of 55 patients were diagnosed with AF (n = 54) or atrial flutter (n = 1), resulting in an overall detection rate of 22.4% (95% confidence interval: 17% to 28%). In the patients in whom AF was found, 27.3%, 50.9%, 69.1%, and 89.1% were detected in the first, third, sixth, and 12th month, respectively. Fifty-one (92.7%) were asymptomatic; the mean ventricular rate was 112.2 ± 30.3 bpm. There was no significant difference in the AF-free survival when patients with high (5–9) and low (2–4) CHA_2_DS_2_-VASc scores were compared (21.0% versus 24.0%; p = 0.58 per log-rank test). However, there were significantly more females in the group with a high CHA_2_DS_2_-VASc score (50.4% versus 32.3%; p < 0.05); additionally, the female subjects (who are much less likely to develop AF at a given age), had approximately half the AF incidence as that of the men (14.9% versus 28.8%; p < 0.05). Following notification that AF was present, caregivers elected to initiate oral anticoagulation (OAC) (which was not protocol driven) in 76.4% of patients, with either a novel oral anticoagulant (n = 38) or warfarin (n = 4). PREDATE AF therefore demonstrated that at least in this one small trial, silent AF appears to be common in patients who do not have a history of AF but in whom high-risk markers for AF are present, and that physicians deem such AF to be important enough to warrant OAC in most of these individuals.

### Prevalence of Subclinical Atrial Fibrillation Using an Implantable Cardiac Monitor in Patients with Cardiovascular Risk Factors (ASSERT-II)

ASSERT-II^33^ was a similarly sized but multicenter trial with a single-arm, open-label protocol that enrolled 273 patients who did not have a history of AF but who had risk markers for it, and who were given an ICM for AF detection. ASSERT-II enrolled patients aged ≥ 65 years who were being cared for in a cardiology/neurology clinic and who had either a CHA_2_DS_2_-VASc score ≥ 2, obstructive sleep apnea, or a body mass index > 30; and either a LA volume ≥ 58 mL or a LA diameter ≥ 4.4 cm, or a serum N-terminal prohormone of brain natriuretic peptide ≥ 290 pg/mL. Two hundred fifty-six patients received the ICM, four died, and 252 completed a minimum of nine and a maximum of 16.3 months of follow-up. Hypertension was present in 73%; additionally, 48% had a prior stroke, transient ischemic attack, or systemic embolism; 9% had a heart failure history; the mean CHA_2_DS_2_-VASc score was 4.14; the mean age was 74 years; and 34% were female. The primary endpoint was subclinical AF (SCAF) lasting at least five minutes. The incidence of AF ≥ five minutes was 34.4% per person-year. At 18 months, AF lasting ≥ 30 minutes, ≥ six hours, and ≥ 24 hours was 21.8%, 7.1%, and 2.7%, respectively. Using baseline characteristics, of the subgroups examined, a LA volume of at least 73.5 mL was a predictor of AF (hazard ratio (HR): 1.85; p = 0.015). AF was also more common in older patients [mean age: 75.3 in those with AF, 73.1 in those without AF (HR: 1.31; p = 0.008)]. Interestingly, the systolic blood pressure (140 mmHg versus 135 mmHg), history of heart failure (11.4% versus 3.3%), the presence of diabetes (28.9% versus 17.8%), and the presence of vascular disease (36.1% versus 24.4%) were higher in those patients without versus in those with AF detected. For the presence of AF ≥ five minutes, only LA volume remained significantly different. A stroke history was no more common in those with AF detected than in those without. Similar to PREDATE AF, the ASSERT-II investigators concluded that SCAF is common not only in patients with pacemakers, but also more broadly in elderly individuals with specific risk markers.

### A prospective study of previously undiagnosed atrial fibrillation as detected by an ICM in high-risk patients

REVEAL AF^34^ was a prospective, single-arm, open-label, multicenter trial that assessed 446 patients who did not have a history of AF but who had high-risk markers for it. Three hundred ninety-four of these individuals underwent ICM insertion for silent AF, with 385 being eligible for analysis. Fifty-two of the 446 patients were excluded, primarily due to an inability to meet the inclusion/exclusion criteria, or at the patient’s request. Nine of the 394 were further excluded from analysis because of an inclusion criteria violation, use of an antiarrhythmic drug, or no post-insertion data. Inclusion criteria included a CHADS_2_ score of ≥ 3 or a score of 2, plus at least one of the following: coronary artery disease, renal impairment (glomerular filtration rate: 30 to 60 mL/min), sleep apnea, or chronic obstructive pulmonary disease. At least 70 patients were required for each CHADS_2_ group (2, 3, ≥ 4, respectively). Device transmissions were made monthly and in-office follow-up visits occurred every six months for a minimum of 18 and a maximum of 30 months. All included patients were required to have a minimum of 24 hours of external ECG monitoring within 90 days prior to enrollment or device insertion in which no AF was detected. This inclusion criterion was unique to REVEAL AF and was not used in either PREDATE AF or ASSERT-II. The primary outcome was the incidence of adjudicated AF ≥ six minutes at 18 months. Secondary and additional exploratory objectives included predictors of AF, physician actions (OAC prescription in particular) in response to AF detection, AF incidence at additional time points from 30 days to 30 months, a comparison of AF incidence among the CHADS_2_ subgroups, and the median time from implant to AF detection.

In REVEAL AF, the mean age was 71.6 years, hypertension was present in 93.7%, 20.6% had a history of heart failure, 59.1% had coronary artery disease, 62.9% had diabetes, 49% were women, the mean CHADS_2_ score was 2.9, and the mean CHA_2_DS_2_-VASc score was 4.4.

The primary endpoint in REVEAL AF occurred in 29.3% at 18 months. Of these 128 subjects, at least one AF episode was ≥ 24 hours in 10.2%. Adjudicated AF of ≥ six minutes was also present in 40.0% at 30 months. It was 6.2% at 30 days. There were no significant differences in these events across the three CHADS_2_ subgroups. The median time to detection was 123 days. Hence, AF would not have been detected in this trial in over three-quarters of the patients had monitoring been limited to one month. Similarly, the rates of AF detected in both PREDATE AF and ASSERT-II were also quite low in the first month. Only age and body mass index were predictors of AF out of all the baseline demographic criteria assessed. Among the patients who met the primary endpoint, 56.3% were prescribed an OAC. As in PREDATE AF, this action was not protocol driven. Thirteen patients died following enrollment, but no deaths were related to the device in any way. Most of the patients had one or more non-specific symptoms at baseline (approximately 10% had none), but there were no differences in outcome events in those with versus those without symptoms.^32,35^ This finding is not really surprising, since symptoms have been proven to have a poor correlation with AF in many studies.^36–39^

Thus, REVEAL AF, a somewhat larger and longer trial than PREDATE AF or ASSERT-II, confirmed a significant incidence of previously undiagnosed AF in a group of patients identified demographically to be at-risk for both AF and stroke, in whom a history of AF was absent and no previously implanted pacemaker/ICD was present. The at-risk populations studied in REVEAL AF, PREDATE AF, and ASSERT-II are a common group of patients encountered in clinical practice. The willingness of physicians to have their patients enroll in such trials and their high rate of initiation of OAC therapy when AF is detected reveals their now-evident concern regarding the presence of silent AF. However, the initiation of unblended, open-label anticoagulation in these trials precludes the assessment of the risk of stroke or the certain benefit of anticoagulation in them.

### Role of possible treatments: ARTESiA and NOAH-AFNET 6

Accordingly, it is unproven whether OAC treatment of silent AF episodes, especially if relatively short, can significantly reduce the risk of thromboembolism. Two trials, ARTESiA (NCT01938248) and NOAH-AFNET 6 (NCT02618577) are underway to assess the potential role of OAC in patients with device-detected AF. ARTESiA is a prospective, randomized, double-blind study designed to determine if treatment with standard-dose apixaban versus aspirin at 81 mg/day will reduce the risk of ischemic stroke and systemic embolism in patients with device-detected silent AF of at least six minutes, but not of > 24 hours, in duration, plus a CHA_2_DS_2_-VASc score of at least 4. NOAH-AFNET 6 is an investigator-initiated, prospective, parallel-group, double-blind, randomized multicenter trial assessing the prevalence of stroke, symbolic embolism, and death in patients aged at least 65 years old who have a CHA_2_DS_2_-VASc score of at least 2 and pacemaker/ICD detected silent AF treated with edoxaban versus aspirin at 100 mg/day. All of these trials are expected to be completed between 2019 and 2021.

## We are not alone

**Figure 2:**
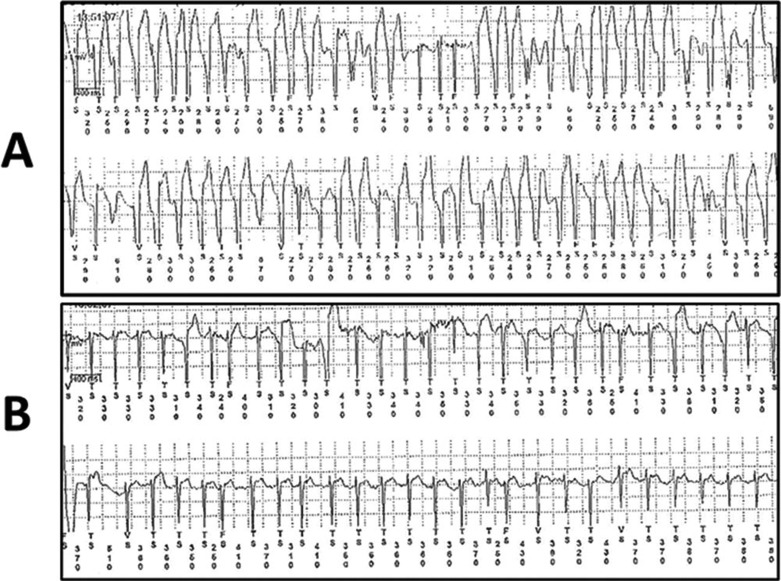
Silent AF documented with an ILR. Two recordings obtained with an ILR in a chimpanzee are visible. At the time of this recording, this chimpanzee was being cared for at the non-profit organization Save The Chimps’ sanctuary in Ft. Pierce, FL, USA. These recordings are courtesy of Save the Chimps Senior Veterinarian Dr. Jocelyn Bezner, who is studying cardiomyopathy in male chimpanzees as part of the Great Apes Project. **A:** Rapid AF with aberrant conduction. **B:** Atrial flutter with variable conduction but a slower ventricular response.

Finally, we physicians who care for our fellow humans are not the only ones struggling with silent AF. Our veterinarian colleagues do as well. Clinically meaningful silent AF is not just a disorder of humans: it occurs in many species, including in chimpanzees, our genetically closest and now endangered relatives. Importantly, 40% of male chimpanzees in captivity die of cardiac disease—a dilated, fibrotic, and sometimes fat-replaced cardiomyopathy. Some have had rapid AF demonstrated. Whether this is a consequence of the cardiomyopathy or the cause of it has not yet been determined. Certainly the chimps do not complain of symptoms, yet the AF is serious. An example, obtained with an ILR, and hence pertinent to the above discussion, is shown in **Figure 2**.

## Closing thoughts

In conclusion, the final points are to be remembered:

1. Silent AF appears to be relatively common—at least in patients with high-risk factors for AF and stroke, and in patients with electrical disorders that require implantation of a therapeutic electrical device.
2. Silent AF likely carries a risk of thromboembolism when it occurs in the setting of stroke risk factors, especially if the burden is high and/or the comorbidities are marked. However, the definitive answer as to the benefit of initiating OAC in patients with device-detected silent AF will remain unsettled for the present. Nonetheless, the findings reported in the Find-AF_randomized_ trial discussed above, plus the current AF guidelines, as well as the physician actions demonstrated in the silent AF trials cited, suggest that the anticipated answer will be yes.
3. The less-settled questions include what will be the threshold for AF burden above which risk-based treatment is justifiable, and who or what are the optimal populations to be screened? Because AF burden and comorbidity severity are synergistic, it is unlikely that a single silent AF duration or frequency will be universal; rather, it will likely vary inversely with the type and severity of the concomitant conditions present in each patient. Additional studies are needed to further elucidate the characteristics of silent AF that are clinically meaningful, and what form optimal therapy will take. Presumably, some of this will come out of the recently established AF-SCREEN international collaboration.^12^

## Figures and Tables

**Table 1:** A Synopsis of PREDATE-AF, ASSERT-II, and REVEAL AF

Trial	Number of Subjects Enrolled	Mean Subject Age	% Female	Primary Endpoint	Event Rate	Average Detection Time
PREDATE-AF^32^	245	74.3 years	41%	AF lasting six minutes or more	22.4% at 451 days	141.3 days
ASSERT-II^33^	273	73.9 years	34%	AF lasting five minutes or more	34.4%/person-year	5.1 months
REVEAL AF^34^	385	71.6 years	48%	AF lasting six minutes or more	29.3% at 18 months	123 days

AF: atrial fibrillation.
